# Ottawa Knee Rule: Investigating Use and Application in a Tertiary Teaching Hospital

**DOI:** 10.7759/cureus.8812

**Published:** 2020-06-24

**Authors:** Abubakr Mohamed, Elkhidir Babikir, Mohamed Kamal Elbashir Mustafa

**Affiliations:** 1 Emergency Medicine, University Hospital Galway, Galway, IRL; 2 Vascular Surgery, University Hospital Galway, Galway, IRL

**Keywords:** ottawa knee rule, knee injuries

## Abstract

Background

Knee injuries are encountered commonly in the emergency departments (EDs) in Ireland. Validated clinical decision rules such as Ottawa knee rule (OKR) can be used in acute knee injury settings to reduce the number of unnecessary radiography. Clinical judgment can be used to distinguish between suspected fractures and non-fractures in many cases; however, radiography is still routinely requested.

Objectives

We evaluated the OKRs in a high-volume tertiary teaching hospital in Ireland to determine whether the rule could be safely used to decide whether patients with acute blunt knee trauma should undergo radiography.

Methods

This was an observational study conducted in the ED over a three-month period in a tertiary referral hospital. A total of 110 patients with acute knee injuries were examined using OKR. Inclusion criteria included patients with acute knee injuries due to blunt trauma or twisting injury and patients with lacerations or contusions. Open fractures and fractures due to penetrating injury were excluded from the study.

Results

Fractures were seen in 12 (13.2%) of the 110 patents who met the inclusion criteria. The OKR predicted all 12 fractures. Sensitivity was 100%, and specificity was 39%.

Conclusions

The OKR is highly sensitive for fracture in this setting and can be safely used to decide whether patients with acute blunt knee trauma should undergo radiography.

## Introduction

The Ottawa knee rules (OKRs) were designed to divide acute knee trauma patients into two groups: patients who are likely to have a clinically significant fracture and need radiography to rule out an injury and patients who have virtually no chance of having an important radiographically detectable bony injury [1-3]. The OKR consists of five components asking whether the patient is 55 years of age or older, has an isolated tenderness of the patella (no bone tenderness of the knee other than at the patella), has tenderness of the head of the fibula, is unable to flex the knee to 90 degrees, and is unable to bear weight both immediately and at the emergency department (ED) for four steps [4]. Patients with at least one positive answer are considered to have positive results for knee fracture and are advised to have radiography. After the development of the rule by Stiell et al. in 1995, the decision aid was validated in several clinical settings [1,2]. In situations where virtually every patient entering an ED with an acute knee injury undergoes radiography, even modest values for specificity may substantially reduce the number of unnecessary radiographs obtained. Therefore, the threshold for the OKR is standardized at high sensitivity and sacrifices specificity to some extent. A systematic review of 4,249 patients in six studies found that fractures can narrowly be excluded in a negative OKR [5,6].

## Materials and methods

This was an observational study conducted in the ED of a tertiary hospital in Ireland over a three-month period. We reviewed all radiology requests and reports of knee radiographies in the ED prospectively over a three-month period (October to December 2019).

Patients were reviewed and their clinical records analyzed to establish the indication for ordering plain radiography. A total of 110 patients were identified. Patient records were reviewed to assess whether any of the Ottawa knee criteria were fulfilled when plain radiography was being ordered. The data collected was analyzed for diagnostic accuracy (sensitivity, specificity, positive predictive value, and negative predictive value) of the OKR.

## Results

**Figure 1:**
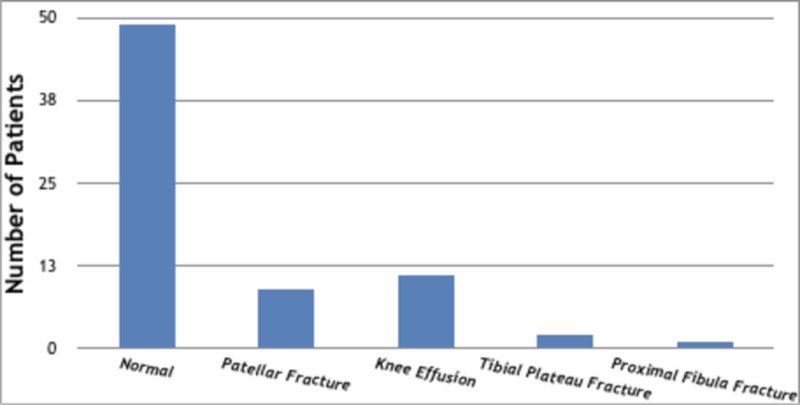
Acute knee trauma diagnosis.

The study included 110 patients between 2 and 88 years of age (mean: 41.5 years). The gender distribution was equal, with a female-to-male ratio of 1:1 (Table 1). The majority of presentation was in the age group of 10 to 19 years (19.09%) followed by the age group of 60 to 69 years (17.27%). Undiagnosed knee injury was the most common diagnosis (44.55%; n = 49) followed by knee fractures (10.9%). Knee effusion accounted for 10% of the injuries (Figure 1).

**Table 1:** Acute knee trauma patient demographics and injury description.

Characteristics	Values
Age (years)	
Mean (median)	43 (41.5)
Interquartile range	47
Gender (%)	
Male	50% (n = 55)
Female	50% (n = 55)
Mechanism of injury (%)	
Fall	75%
Twisting	8.33%
Direct blow	9.72%

**Figure 2:**
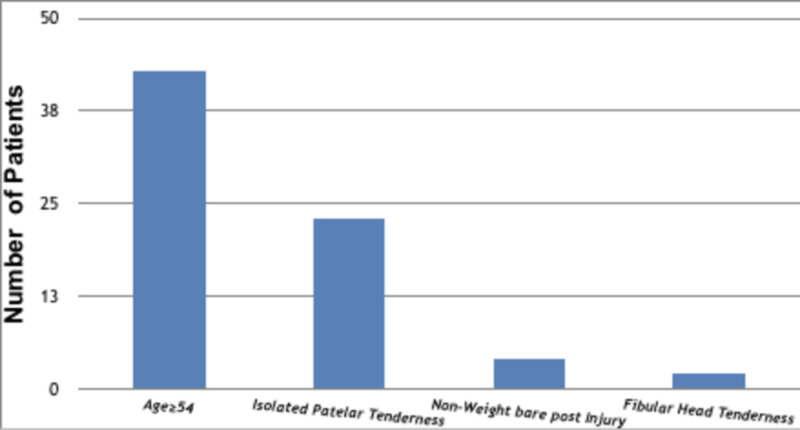
Ottawa knee rule fulfillment.

Each patient’s radiography request was assessed to determine whether the Ottawa criteria were fulfilled. Of 110 of request forms, 72 (65%) were justified with enough written information to warrant an X-ray according to the OKR. The most common reason for fulfilling the Ottawa criteria was the patient age (59.72%) followed by patellar tenderness (31.94%), inability to bear weight (5.56%), and fibular head tenderness (2.78%) (Figure 2). Limited knee flexion to 90 degree was not present in all knee radiography requests. Noncompliant knee radiography had insufficient information in some cases to see whether radiography was appropriate or not, and patients who were not referred for radiography were excluded and they may have been compliant.

Of the patients who met the Ottawa knee criteria, 75% had a fall as their mechanism of injury followed by blunt trauma (9.72%), twisting (8.33%), and unknown mechanism (6.94%) (Table 1).

Twelve patients from the study group were diagnosed with a fracture. All 12 fulfilled the OKR. Using the Ottawa rules for knee radiography, 38 radiographs (34.55%) could have been avoided without missing a fracture. The various parameters of diagnostic accuracy for the rules are shown in Table 2. However, OKR was found to have a relatively low positive predictive value of 61% and a negative predictive value of 100%.

**Table 2:** Diagnostic accuracy of the Ottawa knee rules.

Parameters	Values (%)
Sensitivity	100%
Specificity	39%
Positive predictive value	61%
Negative predictive	100%

## Discussion

Two of the most important attributes of good clinical prediction rules for fracture are high sensitivity and external validity [2,3]. Clinicians are uneasy using prediction rules that are not perfectly sensitive, that is, do not detect all cases partly because of medicolegal fears.

Stiell et al. recognized this preference when deriving the OKRs: a second rule with a much higher specificity (allowing more substantial reduction in radiography) could have been devised but only at the expense of lower sensitivity. They believe that this decreased sensitivity would be unacceptable to patients and physicians because a small number of fractures would be missed [1]. A later study was performed to validate the OKR, and prospective validation analyzing 1,096 patients found it to be 100% sensitive for identifying knee fractures [1,2]. The decision rule was interpreted correctly 96% of the time, and, when applied, the probability of missing a fracture was zero [1]. The decision rule was 100% sensitive for identifying fractures in patients aged 18 years who were not referred from other hospitals, returned for reassessment, had knee injuries for seven days, or had isolated skin lesions.

This study shows the rules to be an eﬀective tool in deciding which patients with isolated acute knee injury do not require knee radiography when used by doctors in ED with. Also, it shows that ED clinicians do not seem to be adherent to using any specific criteria for ordering plain radiography when acutely reviewing knee injuries. Previous studies have shown a reduction in the number of radiographs requested after the introduction of the OKR [4-8].

In our study, the Ottawa rule was found to have a sensitivity of 100% and a specificity of 39%. If the OKR was followed exclusively, 38 radiographs could have been avoided, with no missed fractures. All of the 12 fractures in this study were identified using radiography, and no further investigation were needed.

It is important for radiologists and emergency medicine physicians to understand that patients can have radiography safely deferred (as used by Stiell et al.). Patients whose symptoms resolve have no need for radiography, but in patients with persistent pain, radiography may be necessary. Patients should be instructed to return for further evaluation, including possible radiography, if symptoms do not improve in five to seven days.

One of the limitations of our study is that we did not follow up with the patients to see if further imaging investigations were obtained or who did not receive radiography at the time of ED presentation, and it is possible that not all patients with fractures were identified. Another limitation is the sample size. Relatively small numbers of patients were included, and accuracy might have been overestimated. Additionally, the definition of any criterion might be differently comprehended by different physicians and advanced nurse practitioners (ANPs).

## Conclusions

In summary, we found the OKRs to have high sensitivity and a reliable screening technique in patients with acute blunt knee trauma. However, compliance with the OKR among academic ED healthcare providers is poor. Improving compliance will require a comprehensive approach involving both education (of physicians and ANPs) and system interventions.
